# Color, pH, microbiological, and sensory quality of crickets (*Gryllus bimaculatus*) flour preserved with ginger and garlic extracts

**DOI:** 10.1002/fsn3.3262

**Published:** 2023-03-02

**Authors:** Jolly Oder Akullo, Beatrice N. Kiage‐Mokua, Dorothy Nakimbugwe, Jeremiah Ng'ang'a, John Kinyuru

**Affiliations:** ^1^ Department of Animal Production and Management, Faculty of Agriculture and Animal Sciences Busitema University Soroti Uganda; ^2^ Department of Human Nutrition Sciences, School of Food and Nutrition Sciences Jomo Kenyatta University of Agriculture and Technology Nairobi Kenya; ^3^ Department of Food Technology and Nutrition, School of Food Technology, Nutrition and Bio‐engineering Makerere University Kampala Uganda; ^4^ Department of Food Science and Technology, School of Food and Nutrition Sciences Jomo Kenyatta University of Agriculture and Technology Nairobi Kenya; ^5^ African Institute for Capacity Development Nairobi Kenya

**Keywords:** acceptability, cricket flour, insect‐based foods, spice extracts, storage

## Abstract

Although spices have been used in food for centuries, little is known about their use to preserve insect‐based foods. This study assessed the flour produced from blanched crickets treated with extracts of either ginger, garlic or both at a ratio of 1:4 (v/w) for color, pH, microbiological profile, sensory quality, and acceptability. Sodium benzoate treated and untreated cricket flour was used as positive and negative controls, respectively. The flour was stored at ambient conditions and analyzed on 0, 30, and 60 days of storage. The pH, moisture content and color change increased during storage but remained within acceptable limits. The total microbial count, yeast and molds significantly decreased with storage duration (*p* ˂ .05), while fecal coliforms and *Escherichia coli* were not detected in any of the samples. At the end of the 60‐day storage period, cricket flour treated with sodium benzoate and garlic extracts both had a significantly lowest population of yeast and molds (1.91 log cfu/g). On five point hedonic scale (1. Dislike extremely and 5. Like extremely), color (3.84 ± 0.86–2.55 ± 0.99), aroma (3.59 ± 1.09–2.40 ± 1.01), texture (4.11 ± 0.97–3.11 ± 0.97) and overall acceptability (3.77 ± 0.64–2.83 ± 1.01) sensory scores were all significantly high on day 0 and low on day 60 of storage, respectively. The study concluded that preserving crickets with garlic extracts significantly reduced the population of yeast and molds. Cricket flours were microbiologically safe and acceptable to consumers. Therefore, storage of cricket flour preserved with garlic and ginger extracts for longer periods is recommended. In addition, utilization of the preserved flour as an ingredient in different food applications is recommended to determine its suitability and sensory acceptability.

## Graphical abstract

At the end of the 60‐day storage period, cricket flour treated with sodium benzoate and fresh garlic extracts had the lowest microbial counts including yeast and molds. The pH, moisture content and color change remained within acceptable limits for both treated and untreated cricket flour. Consumers liked both the spice‐treated and control cricket flour samples, although acceptability declined with prolonged storage.

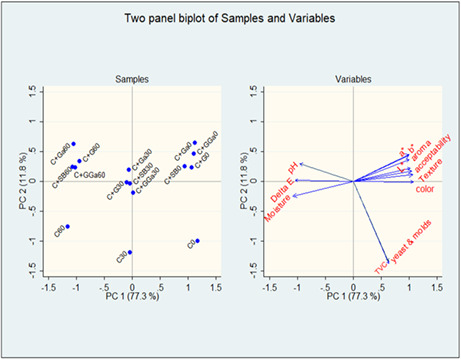

## 1 INTRODUCTION

Many societies around the world consume insects as an essential component of their diet (Raubenheimer & Rothman, 2013). In terms of nutrition, insects are an excellent source of proteins, fat, vitamins, and minerals (Akullo et al., 2018; Maiyo et al., 2022). In Africa, 212 insect species from eight orders are consumed as food (Hlongwane et al., 2020). In East Africa, widely consumed edible insects include winged termites (*Macrotermes* spp.), grasshoppers (*Ruspolia* spp.) and different species of crickets (Kinyuru et al., 2013; Maiyo et al., 2022; Malinga et al., 2018). Among the different edible insects, crickets (*Gryllus bimaculatus*) are a promising insect species since they have high nutritional quality and they can also be successfully reared under farm conditions (Kinyuru & Kipkoech, 2018; Ng'ang'a et al., 2020; Sorjonen et al., 2019). The current world population's rapid growth and low food production has caused a shortage of protein supplies and widespread malnutrition. Among the possible approaches to closing the gap between current and future levels of food production and consumption are: exploiting new, unconventional food sources such as insects; as well as enriching low‐quality meals with high‐quality protein sources such as those derived from insects. However, seasonality and perishability are major barriers to utilizing insects as food (Ayieko et al., 2010), in addition to considerable post‐harvest losses. Moreover, insects are a highly perishable food due to their high water activity, free amino acids, polyunsaturated fatty acids, and nutritional value. The biological processes that result in insect spoilage include the oxidation of lipids, the activity of their intrinsic enzymes, and their metabolic processes. These biological processes affect the physical, organoleptic, and nutritional qualities of insect‐based food products (Mokhtar et al., 2014). The addition of antioxidants is the most effective way to prevent oxidation and preserve insects, and currently, using natural plant extracts as preservatives is a popular trend because consumers are concerned about the safety of synthetic antioxidants and chemical preservatives (Shah & Mir, 2022). Furthermore, some herbs and spices, including ginger and garlic, have been utilized to successfully preserve other perishable foods such as fish and meat, respectively (Brewer, 2011). A number of studies have also reported on the food preservation potential of ginger and garlic (Akintobi et al., 2013; Indu et al., 2006; Khashan, 2014; Mohammed et al., 2019).

The preserved products were reported to have good nutritional and sensory quality owing to the action of the antioxidant and antimicrobial components of the extracts. Besides these, spices have been used to change the physical appearance of food, hence influencing its consumer acceptability. For instance, pepper and turmeric change the color, appearance, and taste of most foods, with many health benefits (Pop et al., 2019). The influence of several natural antioxidants on the sensory qualities of beef patties during storage was examined (Mokhtar et al., 2014); revealing significantly higher sensory scores for odor, color, taste, and overall acceptability than the control. However, the same study showed no substantial difference in the textures of treated and untreated samples. The color parameters of the raw pork samples treated with spice extracts changed slightly during storage, but the control samples exhibited significant changes (Shan et al., 2009). Procyanidin treatment resulted in reduced pH and lightness values of pork patties, while the redness was enhanced in contrast to the untreated controls (Jeong et al., 2015).

According to Goyal et al. (2017), lengthy storage can completely destroy the quality of flour. The main issues reducing the shelf life of flour are microbiological growth, color change, and oxidative rancidity. Previous research suggests that crickets are contaminated with both harmful and spoilage micro‐organisms (Fröhling et al., 2020; Grispoldi et al., 2021). Furthermore, the sensory acceptability of a traditionally processed meat‐based product was marginally lowered after storage for 12 weeks (Mgbemere et al., 2011). Sensory acceptability has become a vital part of food production. A consumer's first impression of a food product, which is primarily determined by color, is what causes them to accept or reject it (Pathare et al., 2013). As much as plant extracts have been used to preserve food as aforementioned, their use to preserve insects has not been attempted previously, and it is not known whether insect preservation with plant extracts can extend the shelf life and improve the sensory acceptability of the resultant insect food products. In this study, we used extracts from garlic and ginger to preserve raw crickets, which were later processed into flour. The storage quality (changes in color, moisture, pH, microbial profile, and sensory acceptability) of the flour was evaluated during 60 days of storage. The goal was to provide an affordable, safe, and acceptable alternative for the preservation of insects, making insect flour available for a wide range of food applications.

## 2 MATERIALS AND METHODS

### 2.1 Collection of spices and preparation of extracts

Fresh spices (garlic cloves and ginger rhizomes) were purchased from a local market in Uganda and transported in a cool box to the laboratory at the Jomo Kenyatta University of Agriculture and Technology (JKUAT), for processing and extraction. Samples were sorted to remove bad quality, soaked in water for 30 min, washed and rinsed under running water; with care to avoid any tissue damage. Subsequently, the skin was peeled prior to crushing/grating for extraction according to previously established procedures (Tanvir et al., 2017). Forty (40) g of ginger, garlic, and ginger + garlic (20 g each) were transferred into individual 500 mL conical flasks, followed by the addition of 200 mL of absolute ethanol. The flasks were wrapped in aluminum foil to limit the reaction of their content with light and shaken for 24 h at 300 rpm on a mechanical shaker (KS 250 basic, Ika Labortechnik‐Japan). Afterwards, the solution was filtered using Whatman filter paper No 1; and the filtrate was concentrated using rotary evaporation at 50°C. Each of the concentrated extracts was re‐dissolved in distilled water to make 200 mL for the subsequent treatment of crickets.

### 2.2 Collection, treatment and processing of cricket flour samples

Adult live crickets (*G. bimaculatus*) were obtained from insectPro farm, which is located in Limuru, Kenya. Prior to harvesting, crickets were fasted for 48 h while being given only water to cleanse their guts of the ingesta. The crickets were transported to the laboratory in aerated boxes and inactivated by freezing them at −20°C overnight. Frozen crickets were allowed to thaw at room temperature for 2 h. To remove dirt and feed residues, frozen crickets were washed three times in tap water using a cricket to water ratio of 1:3 (w/v) according to previous procedures (Fröhling et al., 2020). Each washing procedure took place for 5 min while being stirred, and then the crickets were removed from the water using a sieve. Washed crickets were blanched by submerging in hot water for 1 min to modify the texture of the samples and inactivate the microorganisms as previously reported (Caparros Megido et al., 2018; Megido et al., 2017). Samples were then divided into five batches of approximately 1000 g each; three batches were chosen randomly and treated with extracts of ginger, garlic, and ginger + garlic at a ratio of 1:4 (v/w). These were labeled as C + G, C + Ga, C + GGa, respectively. The ratio was determined from pre‐trials that considered the quantity of extracts that could be absorbed in the crickets with little excess solution for draining. The other two batches received 0.1% sodium benzoate (C + SB) as a positive control and distilled water (C) as a treatment (a negative control). Each sample was placed in a large container with the extracts or solution, and the container was vigorously swirled for 1 min to mix the samples with extracts/solutions. Following a 30‐min soak in the appropriate solution; any excess was drained off prior to oven drying for 2 h at 105°C (until crisp dry).

High temperature short time drying was adopted to achieve rapid dehydration and avoid the risk of sample decomposition. After drying, the samples were milled into flour and packed in zip‐top low‐density polyethylene bags with a 10 μm thickness. Packed samples were labeled as follows; C + G, C + Ga, C + GGa, C + SB, and C (C = cricket only, G = ginger extracts, Ga = garlic extracts, GGa = ginger and garlic extracts, SB = sodium benzoate). Prior to packaging flour from each treatment was divided in to three parts which were put in separate packages, such that a package was randomly picked for analysis on day 0, 30 or 60. Packages that were opened were not returned to storage. The samples were kept on shelf at ambient conditions; room temperature (23 ± 2°C) and relative humidity (60 ± 2%) and subjected to physicochemical, microbiological, and sensory evaluation at days 0, 30, and 60 storage periods.

### 2.3 Determination of pH, moisture content and cricket flour color

The pH was determined using the method that was previously validated (Kalra, 1995). In brief, the pH was tested by homogenizing 10 g of sample in 100 mL of distilled water. After filtering, the pH was measured in triplicate. Moisture content was determined by weight loss upon drying at 105°C for 2 h, following the procedures of Thiex (2009).

The color of flour was monitored following the method adopted by Wang et al. (2015); using a handheld Minolta color meter (Model CR‐200, Osaka, Japan) according to the International Commission on Illumination (CIE) color space. All measurements were made at ambient temperature upon calibration of the meter using a white plate/surface and reported using the L*, a*, b* color systems. L* represents whiteness/blackness (100 = white and 0 = black); a* represents redness/greenness (a* positive = red, a* negative = green); and b* represents yellowness/blueness (b* positive = yellow, b* negative = blue). Measurement was performed six times on approximately 10 g of flour in a small container. Color changes (ΔL*, Δa*, Δb*) were calculated using the samples at zero as a reference. The total color difference (ΔE*) which shows the extent of color change in parameters between the initial and final color values during storage, was calculated using the equation by (Pathare et al., 2013).

$$∆E*=\sqrt{∆a{*}^{2}+∆b{*}^{2}+∆L{*}^{2}},$$

where, ΔL* = changes in lightness, Δa* = changes in redness/greenness, Δb* = changes in yellowness/blueness, ΔE* = total color change.

### 2.4 Microbiological analysis

The spread plate method was used to measure Total Viable Counts (TVC) utilizing Nutrient Agar (NA) as the medium, according to previous procedures (Sanders, 2012). Prior to plating, all tools, solutions, and media were prepared and sterilized in accordance with the Standard Operating Procedures (SOPs) necessary for minimal microbiological contamination. From each of the storage bags, ten (10) g of cricket flour was removed aseptically and placed in a sample bottle containing 90 mL of sterilized physiological saline. This was followed by homogenization, threefold dilutions, and plating on nutrient agar for each of the dilutions. In brief, 0.1 mL of each dilution was aseptically taken using a micropipette onto a pre‐poured, solidified agar plate, and the inoculum was spread using a sterile bent metallic rod. After cooling, the plates were inverted and incubated at 35 ± 2°C for 24 h. In accordance with the methods of Feng et al. (2002), fecal coliforms and *Escherichia coli* were identified by plating on Violet Red Bile Glucose Agar (VRBGR) and incubated at 35 ± 0.5°C for 24 ± 2 h. Total yeasts and molds were determined on Sabouraud Dextrose Agar (SDA) plates incubated at 27°C for 48 h, as recommended (Tournas et al., 2001). Plating was conducted in triplicate and microbial counts were expressed as log colony forming units per gram of sample (log cfu/g).

### 2.5 Sensory evaluation

The sensory/organoleptic properties of flour during storage were evaluated for color, aroma, texture/fineness, and overall acceptability on days 0, 30, and 60 of storage. Sensory quality was evaluated following the principles and practices previously described (Sharif et al., 2017). Processed samples were served to a panel and rated for the mentioned parameters using the hedonic test on a 5‐point scale (1 = Dislike extremely, 2 = Dislike moderately, 3 = neither like nor dislike, 4 = like it moderately, 5 = like it extremely). Each testing session consisted of 60 untrained panelists, both males and females, randomly recruited from among employees and students from the School of Food and Nutrition Sciences, JKUAT. Following the briefing, coded samples were given to each participant at random for rating.

### 2.6 Statistical analysis

Data was entered in Microsoft Excel for storage and analyzed using STATA version 12 (StataCorp LP, Texas, USA). Analysis of Variance (ANOVA) was performed and and means were separated using Bonferroni adjustment at 95% confidence level. Results were reported as mean ± standard deviations. PCA was applied after the Two way ANOVA to a set of data that was obtained from the color, moisture, pH, microbiological analysis, and sensory evaluation of samples from various treatments and storage intervals. The original data was transformed into a smaller set of linear combinations called principal components (PC). Two PCs were chosen, and their combination described the best treatment and storage time.

## 3 RESULTS

### 3.1 Cricket flour color, moisture content and pH

There was a significant interaction between sample treatment and storage duration for Total color change and Lightness values, as shown in Table 1. As the samples were stored, their lightness (L*) values declined from day 0 to day 60. For both the treated and untreated samples, lightness at day 0 was significantly higher than at days 30 and 60 in storage (*p* ˂ .05). However, lightness values across samples at the same stage of storage did not differ significantly. Measurement for redness/greenness (a*) did not vary significantly among samples (*p* > .05). The highest value was observed on day 0, while the lowest value was on day 60 for all the samples. The flour from cricket preserved with garlic and garlic mixed with ginger had the highest values at day 0 (a* = 4.97 ± 0.12 and 4.73 ± 0.29, respectively). In the garlic/ginger‐treated, sodium benzoate‐treated, and untreated samples, the measurement for yellowness/blueness (b*) of the cricket flour samples varied significantly at day 0 and day 30 of storage (*p* ˂ .05). The total color change (Δ*E*) of samples from the initial storage to the final storage period differed significantly (*p* ˂ .05). The highest color change was noted at day 60 of storage in the untreated samples (Δ*E* = 11.71 ± 0.55). The color change between day 0 and 30 was minimal among samples, ranging from 2.09 ± 1.01 to 2.87 ± 1.09 in flour preserved with garlic and untreated flour.

**TABLE 1:** Color changes during storage of cricket flour preserved with ginger and garlic extracts.

Sample (flour)	Storage (days)	L*	a*	b*	Δ*E*
C + G	0	31.90 ± 0.20^d^	4.70 ± 0.62^bc^	16.10 ± 0.44^b^	0.00 ± 0.00^a^
	30	26.67 ± 0.32^bc^	3.60 ± 0.17^abc^	10.00 ± 1.31^a^	2.29 ± 0.19^b^
	60	25.07 ± 0.06^abc^	3.23 ± 1.12^abc^	8.97 ± 1.10^a^	10.05 ± 0.72^cd^
C + Ga	0	32.00 ± 0.26^d^	4.97 ± 0.12^c^	16.30 ± 0.53^b^	0.00 ± 0.00^a^
	30	25.43 ± 0.21^bc^	4.30 ± 0.96^abc^	9.53 ± 1.05^a^	2.09 ± 1.01^ab^
	60	26.07 ± 0.75^bc^	3.73 ± 0.51^abc^	10.23 ± 1.05^a^	8.59 ± 1.06^c^
C + GGa	0	31.20 ± 0.79^d^	4.73 ± 0.29^bc^	16.57 ± 0.61^b^	0.00 ± 0.00^a^
	30	26.83 ± 0.76c	4.00 ± 0.95^abc^	10.27 ± 0.85^a^	2.27 ± 1.23^b^
	60	25.80 ± 1.54^bc^	2.63 ± 0.51^a^	9.67 ± 0.23^a^	9.04 ± 0.80^c^
C + SB	0	31.07 ± 0.31^d^	4.40 ± 0.26^abc^	14.40 ± 0.46^b^	0.00 ± 0.00^a^
	30	25.57 ± 0.15^bc^	3.80 ± 0.26^abc^	10.00 ± 0.95^a^	2.18 ± 0.45^ab^
	60	24.67 ± 0.12^ab^	2.87 ± 0.61^ab^	8.50 ± 0.26^a^	8.86 ± 0.35^c^
C	0	32.90 ± 0.52d	4.33 ± 0.31^abc^	15.07 ± 0.31^b^	0.00 ± 0.00^a^
	30	25.97 ± 1.44^bc^	3.47 ± 0.32^abc^	9.00 ± 1.15^a^	2.87 ± 1.09^b^
	60	23.30 ± 0.40^a^	2.83 ± 0.55^ab^	8.63 ± 1.36^a^	11.71 ± 0.55^d^
*p* value		<.001	.905	.365	.018

*Note*: Values = mean ± standard deviation (*n* = 6); values with different superscripts along the column are significantly different (*p* ˂ .05).

Abbreviations: a*, redness/greenness; b*, yellowness/blueness; C, cricket; G, ginger; Ga, garlic; GGa, ginger + garlic; L*, lightness; SB, sodium benzoate; Δ*E*, total color change.

Table 2 shows the changes in moisture content and pH of samples during storage. The moisture content of the samples increased with the increase in the number of days that the samples were in storage. However, the rise was more significant (*p* ˂ .05) across the various storage stages than it was within samples. The moisture level ranged from 1.21 ± 0.32% to 1.48 ± 0.11% on day 0; by day 60 of storage, it was in the range of 2.82 ± 0.08%–3.02 ± 0.46%. The pH values increased from day 0 to day 60 during storage, showing a significant difference between samples and storage time (*p* ˂ .05). At day 0, the cricket flour preserved with ginger‐garlic mixed extracts had the lowest pH (6.39 ± 0.00) while the cricket flour treated with sodium benzoate had the highest (6.43 ± 0.00). The sodium benzoate‐preserved samples had the lowest pH at the end of the storage period, whereas the ginger‐preserved cricket flour had the highest pH (6.75 ± 0.01 and 6.90 ± 0.03, respectively).

**TABLE 2:** Changes in moisture and pH of cricket flour preserved with extracts of ginger and garlic.

Sample	Storage (days)	Moisture content	pH
C + G	0	1.47 ± 0.00^a^	6.42 ± 0.01^bcd^
	30	2.34 ± 0.33^ab^	6.46 ± 0.01^e^
	60	2.9 ± 0.4^b^	6.90 ± 0.03^i^
C + Ga	0	1.21 ± 0.32^a^	6.40 ± 0.01^ab^
	30	1.90 ± 0.16^ab^	6.41 ± 0.01^abc^
	60	2.9 ± 0.4^b^	6.85 ± 0.01^h^
C + GGa	0	1.42 ± 0.51^a^	6.39 ± 0.00^a^
	30	2.14 ± 0.27^ab^	6.45 ± 0.01^de^
	60	2.9 ± 0.3^b^	6.84 ± 0.01^h^
C + SB	0	1.44 ± 0.93^a^	6.45 ± 0.01^de^
	30	2.33 ± 0.65^ab^	6.43 ± 0.00^cde^
	60	2.8 ± 0.1^b^	6.75 ± 0.01^f^
C	0	1.48 ± 0.11^a^	6.42 ± 0.01^abc^
	30	2.53 ± 0.14^ab^	6.43 ± 0.01^cde^
	60	3.0 ± 0.5^b^	6.81 ± 0.01^g^
*p* value		.9714	<.001

*Note*: Values = mean (*n* = 3) ± standard deviation. Values with different superscripts along the column are significantly different (*p* ˂ .05). *p* values are showing the interaction effect of treatment and storage.

Abbreviations: C, cricket flour; G, ginger; Ga, garlic; GGa, ginger + garlic; SB, sodium benzoate.

### 3.2 Microbiological analysis

While *E. coli* and fecal coliforms were undetected, the total microbial load and yeast and mold counts decreased with storage duration (Table 3). The TVC of the cricket flour preserved with garlic extracts (1.91 log cfu/g) was the lowest at the end of the storage period. The interaction between sample treatment and storage duration was not significant (*p* > .05). Among the samples and the storage period, a significant decline in the population of yeast and molds was observed (*p* ˂ .05); with the untreated samples having the highest population on days 0 and 30 of storage (4.66 ± 0.01 and 3.89 ± 0.10 log cfu/g, respectively). At the end of the 60‐day storage period, cricket flour treated with sodium benzoate and garlic extracts had the significantly lowest population of yeast and molds (1.91 log cfu/g).

**TABLE 3:** Effect of storage duration on microbial profile of cricket flour preserved with ginger and garlic extracts.

Sample (flour)	Storage (days)	Total plate count (log cfug)	Fecal coliforms	*Escherichia coli*	Yeast and molds (log cfug)
C + G	0	3.62 ± 0.02^bcd^	ND	ND	3.03 ± 0.11^c^
	30	3.19 ± 0.22^bcd^	ND	ND	2.30 ± 0.12^abc^
	60	2.50 ± 0.16^ab^	ND	ND	2.06 ± 0.21^ab^
C + Ga	0	3.07 ± 0.29^abc^	ND	ND	2.65 ± 0.06^abc^
	30	2.54 ± 0.21^abc^	ND	ND	2.63 ± 0.34^abc^
	60	1.91 ± 0.00^a^	ND	ND	1.91 ± 0.00^a^
C + GGa	0	3.18 ± 0.13^bcd^	ND	ND	2.93 ± 0.09^c^
	30	3.37 ± 0.76^bcd^	ND	ND	2.81 ± 0.08^bc^
	60	2.45 ± 0.34^ab^	ND	ND	2.06 ± 0.21^ab^
C + SB	0	3.72 ± 0.02^cde^	ND	ND	2.56 ± .07^abc^
	30	3.34 ± 0.07^abc^	ND	ND	2.26 ± 0.49^abc^
	60	2.56 ± 0.07^abc^	ND	ND	1.91 ± 0.00^a^
C	0	4.80 ± 0.04^e^	ND	ND	4.66 ± 0.01^d^
	30	4.23 ± 0.05^de^	ND	ND	3.89 ± 0.10^d^
	60	2.74 + 0.18 ^abc^	ND	ND	2.91 ± 0.00^c^
*p* value		.088			<.001

*Note*: Values = mean (*n* = 3) ± standard deviation. Values with different superscripts along the column are significantly different (*p* ˂ .05). *p* values are the interaction effect of treatment and storage.

Abbreviations: C, control; C, cricket flour; G, ginger; Ga, garlic; GGa, ginger + garlic; ND, not detected; SB, sodium benzoate.

### 3.3 Sensory evaluation

Sensory attributes of the treated and untreated cricket flour are shown in Table 4. The color of the cricket flour was significantly different among samples during storage (*p* ˂ 0.05). The rating for color decreased with an increase in the number of days in storage. Similarly, the aroma of the treated and untreated cricket flour decreased significantly with the length of time in storage (*p* ˂ .05); and the aroma was the least rated of all the parameters. Texture was rated higher than other sensory attributes in all the samples. The sodium benzoate and spice‐treated samples were all accepted by the panelist up to 60 days of storage with differing levels of acceptability (*p* ˂ .05); the untreated samples were not recommended at day 60 of storage, with a score of 2.83 ± 1.01.

**TABLE 4:** Effect of treatment and storage on sensory attributes and acceptability of cricket flour.

Sample (flour)	Storage (days)	Color	Aroma	Texture	Acceptability
C + G	0	3.67 ± 0.94^c^	3.59 ± 1.09^c^	3.89 ± 0.78^cd^	3.77 ± 0.64^d^
	30	3.41 ± 0.90^c^	2.74 ± 1.13^ab^	3.55 ± 1.06^bc^	3.36 ± 0.85^bc^
	60	2.74 ± 0.98^ab^	2.75 ± 1.02^ab^	3.30 ± 1.03^ab^	3.25 ± 0.9^bc^
C + Ga	0	3.84 ± 0.86^c^	3.44 ± 1.09^c^	4.07 ± 0.85^de^	3.72 ± 0.76^d^
	30	3.31 ± 0.88^bc^	2.81 ± 0.92^ab^	3.47 ± 1.10^abc^	3.24 ± 0.86^bc^
	60	2.68 ± 0.98^a^	2.40 ± 1.11^a^	3.13 ± 1.14^a^	2.94 ± 1.05^ab^
C + GGa	0	3.72 ± 0.92^c^	3.54 ± 1.03^c^	4.11 ± 0.97^e^	3.77 ± 0.76^d^
	30	3.31 ± 0.80^bc^	2.92 ± 1.16^b^	3.43 ± 0.99^abc^	3.29 ± 0.82^bc^
	60	2.62 ± 0.97^a^	2.69 ± 0.98^a^	3.15 ± 0.89^a^	3.02 ± 1.01^ab^
C + SB	0	3.67 ± 0.85^c^	3.41 ± 1.09^c^	4.10 ± 0.96^e^	3.72 ± 0.80^d^
	30	3.40 ± 0.77^c^	2.72 ± 1.20^ab^	3.59 ± 1.01^bc^	3.50 ± 0.78^c^
	60	2.70 ± 1.01^a^	2.79 ± 0.99^ab^	3.11 ± 0.97^a^	3.08 ± 0.87^b^
C	0	3.72 ± 0.99^c^	3.52 ± 0.99^c^	3.90 ± 0.98^cd^	3.59 ± 0.84^cd^
	30	3.31 ± 0.92^bc^	2.52 ± 1.10^a^	3.52 ± 1.06^bc^	3.43 ± 0.73^c^
	60	2.55 ± 0.99^a^	2.40 ± 1.01^a^	3.26 ± 1.08^ab^	2.83 ± 1.01^a^
*p* value		(.898, .000*) .965	(.189, .000*) .479	(.994, .000*) .710	(.222, .000*) .535

*Note*: Values are the means and standard deviation, *N* = 60, 58, and 53 for day 0, 30, and 60. Values with different superscript along the columns differ significantly (*p* = .05). *p* values in bracket are main effects of sample treatment and storage, respectively, values outside the bracket is the interaction effect.

Abbreviations: C, cricket only; G, ginger extracts; Ga, garlic extracts; GGa, ginger and garlic extracts; SB, sodium benzoate.

* Significant difference among storage duration.

Table 5 shows the sensory evaluation of samples by male and female panelists. In general, the male panelists' color scores were higher ratings than the female panelists'. Male and female panelists significantly differed in their assessments of the color of ginger‐preserved cricket flour at days 0 and 60 of storage (*p* ˂ .05). Additionally, males scored the color of sodium benzoate‐ and garlic‐preserved cricket flour substantially higher than females at day 0 and 60 of storage (*p* ˂ .05), respectively. Male and female panelists significantly differed (*p* ˂ 0.05), in their opinions of the aroma after 30 days of storage of cricket flour preserved with a combination of ginger and garlic extracts, as well as after 60 days of storage of sodium benzoate preserved cricket flour. The texture and overall acceptability ratings of the treated and untreated cricket flour samples did not significantly vary between the genders during storage. However, after 60 days of storage of the sodium benzoate preserved samples, the average overall acceptability of the male panelists (3.47 ± 0.92) was statistically higher than the female panelists (2.92 ± 0.82).

**TABLE 5:** Rating of sensory attributes and acceptability by gender.

Sensory parameter	Storage days	Gender	Samples				
			C + G	C + Ga	C + GGa	C + SB	C
Color	0	Male	4.04 ± 0.52*	4.19 ± 0.62*	3.78 ± 0.89	3.81 ± 0.79	3.78 ± 1.01
		Female	3.38 ± 1.10*	3.56 ± 0.93*	3.68 ± 0.95	3.56 ± 0.89	3.68 ± 0.98
		*p* value	.006	.004	.671	.246	.693
	30	Male	3.55 ± 1.15	3.45 ± 1.05	3.40 ± 0.82	3.55 ± 0.76	3.35 ± 0.99
		Female	3.34 ± 0.75	3.24 ± 0.79	3.26 ± 0.79	3.32 ± 0.77	3.29 ± 0.90
		*p* value	.407	.387	.540	.275	.814
	60	Male	3.27 ± 0.70*	3.00 ± 0.85	3.00 ± 0.76	3.13 ± 0.64*	2.80 ± 1.01
		Female	2.53 ± 1.01*	2.55 ± 1.01	2.47 ± 1.01	2.53 ± 1.08*	2.45 ± 0.98
		*p* value	.012	.134	.073	.048	.247
Aroma	0	Male	3.56 ± 1.09	3.33 ± 1.04	3.56 ± 0.89	3.37 ± 1.08	3.63 ± 0.93
		Female	3.62 ± 1.10	3.53 ± 1.13	3.53 ± 1.10	3.44 ± 1.11	3.44 ± 1.05
		*p* value	.827	.489	.922	.803	.466
	30	Male	2.85 ± 1.23	2.45 ± 0.89	3.10 ± 1.17*	2.75 ± 1.41	2.50 ± 1.05
		Female	2.68 ± 1.09	2.37 ± 0.94	2.47 ± 1.11*	2.71 ± 1.09	2.53 ± 1.13
		*p* value	.601	.750	.049	.906	.932
	60	Male	3.00 ± 0.93	2.87 ± 0.92	2.87 ± 0.92	3.40 ± 1.06*	2.60 ± 0.74
		Female	2.66 ± 1.05	2.79 ± 1.19	2.79 ± 1.19	2.55 ± 0.86*	2.32 ± 1.09
		*p* value	.274	.822	.200	.004	.360
Texture	0	Male	3.96 ± 0.81	4.04 ± 0.90	4.11 ± 0.97	4.22 ± 0.93	3.93 ± 1.24
		Female	3.82 ± 0.76	4.09 ± 0.83	4.12 ± 0.98	4.00 ± 0.98	3.88 ± 0.73
		*p* value	.491	.818	.979	.374	.865
	30	Male	3.55 ± 1.00	3.50 ± 0.89	3.45 ± 0.89	3.551.00±	3.50 ± 0.83
		Female	3.55 ± 1.11	3.45 ± 1.20	3.42 ± 1.06	3.61 ± 1.03	3.53 ± 1.18
		*p* value	.993	.864	.917	.845	.9210
	60	Male	3.33 ± 0.90	3.33 ± 1.05	3.47 ± 0.74	3.27 ± 0.88	3.07 ± 0.88
		Female	3.29 ± 1.09	3.05 ± 1.18	3.03 ± 0.91	3.05 ± 1.01	3.34 ± 1.15
		*P value*	.891	.426	.104	.476	.407
Acceptability	0	Male	3.89 ± 0.51	3.81 ± 0.62	3.81 ± 0.74	3.78 ± 0.58	3.56 ± 0.93
		Female	3.68 ± 0.73	3.65 ± 0.85	3.74 ± 0.79	3.68 ± 0.94	3.62 ± 0.76
		*p* value	.202	.394	.689	.627	.778
	30	Male	3.45 ± 1.00	3.35 ± 1.04	3.35 ± 0.88	3.65 ± 0.67	3.55 ± 0.76
		Female	3.32 ± 0.77	3.18 ± 0.77	3.26 ± 0.79	3.42 ± 0.83	3.37 ± 0.71
		*p* value	.573	.493	.704	.291	.371
	60	Male	3.53 ± 0.92	3.33 ± 0.98	3.33 ± 1.05	3.47 ± 0.92*	2.93 ± 0.96
		Female	3.13 ± 0.88	2.79 ± 1.04	2.89 ± 0.98	2.92 ± 0.82*	2.79 ± 1.04
		*p* value	.143	.088	.156	.039	.646

*Note*: Values are the means and standard deviation, *N* = 60, 58, 53 for day 0, 30, and 60; Males = 27, 20, 15; Females = 34, 38, 38, respectively. Superscripts (*) shows significant difference between gender groups (*p* ˂ .05).

Abbreviations: C, cricket only; Ga, garlic extracts; GGa, ginger and garlic extracts; Gi, ginger extracts; SB, sodium benzoate.

**FIGURE 1:**
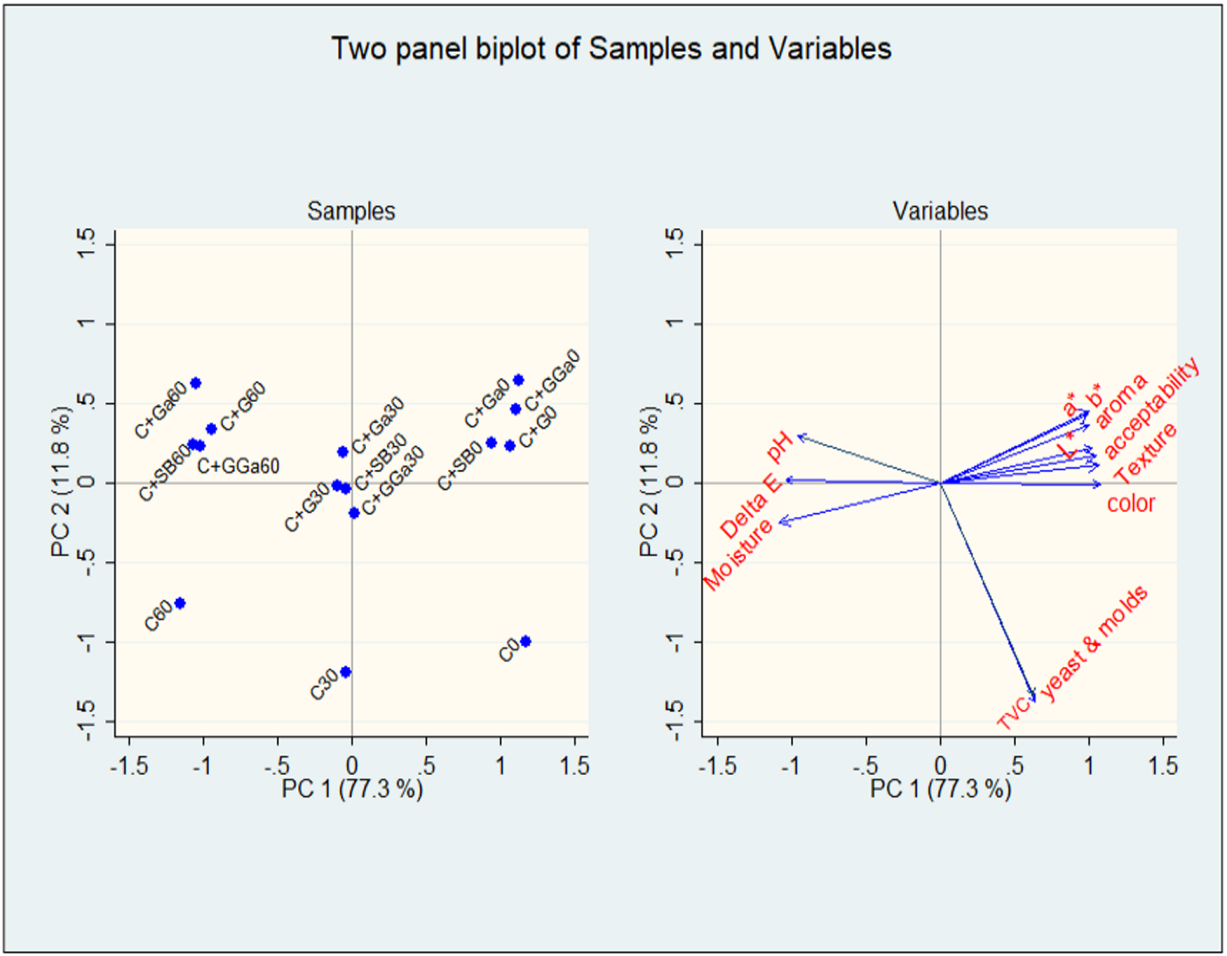
Bi‐plot of loading and sample scores of cricket preserved flours (C, cricket only; G, ginger extracts; Ga, garlic extracts; GGa, ginger and garlic extracts; SB, sodium benzoate). 0, 30, 60 are the days in storage.

There were strong negative correlations seen between the moisture content, pH, and the sensory qualities, while strong positive correlations were seen between the sensory attributes and overall acceptability (Table 6). The eigenvectors of the PCA are shown in Table 7, and the contribution of each PC is shown in Table 8 (Supplementary material 1) Out of the 12 components generated, components 1 and 2 explained most of the variations in the characteristics of the products. The first two‐component accounted for 89.1% of the variance, where principal component 1 (PC 1) had 77.3% of the variance and principal component 2 (PC2) had 11.8% of the variance (Figure 1). The samples were clustered, with the day 0 samples on the PC1's right side; the day 30 samples at the intersection between PC1 and PC2‐negative and positive sides; the day 60 samples on the PC2‐positive side; and the control sample at day 60 on the PC2‐negative side. Color measurements and all sensory attributes are located on the positive side of PC 1, while moisture content and pH are located on the negative and positive sides of PC 2, respectively. On PC1, total viable count, yeast, and molds were all negatively correlated. Day 0 samples had a higher predicted value Table 9 (Supplementary material 2).

**TABLE 6:** Correlation matrix of variables.

Variable	mc	pH	L*	a*	b*	ΔE*	TVC	y & m	Color	Aroma	Texture	Acceptability
Moisture	1.000											
pH	0.835	1.000										
L*	−0.904	−0.627	1.000									
a*	−0.932	−0.784	0.827	1.000								
b*	−0.896	−0.585	0.966	0.847	1.000							
ΔE*	0.912	0.961	−0.780	−0.871	−0.728	1.000						
TVC	−0.440	−0.569	0.457	0.278	0.361	−0.502	1.000					
Yeast & molds	−0.457	−0.534	0.497	0.358	0.393	−0.488	0.880	1.000				
Color	−0.945	−0.932	0.836	0.895	0.799	−0.980	0.544	0.513	1.000			
Aroma	−0.918	−0.624	0.938	0.796	0.933	−0.753	0.399	0.397	0.811	1.000		
Texture	−0.941	−0.789	0.886	0.865	0.898	−0.864	0.539	0.473	0.932	0.873	1.000	
Acceptability	−0.898	−0.806	0.851	0.833	0.832	−0.896	0.486	0.416	0.939	0.863	0.937	1.000

Abbreviations: a*, redness/greenness; b*, yellowness/blueness; L*, lightness; mc, moisture content; ΔE*, total color change.

**TABLE 7:** Principle components (eigenvectors).

Variable	Comp1	Comp2	Comp3	Comp4	Comp5	Comp6	Comp7	Comp8	Comp9	Comp10	Comp11	Comp12
mc	−0.3201	0.1179	0.0197	0.1392	0.1487	0.4373	−0.1367	0.1033	0.3629	0.0263	0.6143	0.3339
pH	−0.2835	−0.1391	0.5394	0.0381	0.2051	0.2116	0.1266	0.233	−0.4076	−0.0507	0.1599	−0.5064
L*	0.3026	−0.1057	0.3768	−0.0992	−0.1464	0.3147	−0.4926	0.1149	−0.3371	−0.0179	−0.1848	0.4702
a*	0.2973	−0.213	−0.1383	−0.5255	0.4052	−0.0291	0.2621	0.5449	0.0853	−0.0785	0.0857	0.1397
b*	0.2942	−0.204	0.3958	−0.0934	0.1993	−0.0255	−0.315	−0.2213	0.6298	0.0731	−0.0545	−0.336
ΔE*	−0.3076	−0.01	0.3755	0.0396	0.2519	−0.2194	0.3017	−0.0432	0.0922	0.526	−0.3138	0.4179
Viable count	0.1886	0.6537	0.1356	0.3469	0.1472	−0.2774	−0.1832	0.4972	0.0765	−0.0453	−0.0782	−0.0696
Yeast	0.1868	0.6341	0.2153	−0.4719	−0.0571	0.2614	0.3112	−0.3446	−0.0348	0.0035	0.0676	−0.0306
Color	0.3197	0.0049	−0.244	0.0657	0.0324	0.1204	−0.1055	0.0282	−0.2186	0.8079	0.2468	−0.2122
Aroma	0.2959	−0.172	0.3438	0.1513	−0.5775	−0.2623	0.3664	0.1378	0.1078	0.0157	0.4029	0.1096
Texture	0.3162	−0.0531	0.0532	0.279	0.5305	−0.2529	0.0549	−0.4323	−0.2982	−0.2185	0.3217	0.2028
Acceptability	0.3096	−0.081	−0.0678	0.4845	0.0779	0.5681	0.4299	0.0379	0.1442	−0.074	−0.3417	−0.0094

Abbreviations: a*, redness/greenness; b*, yellowness/blueness; L*, lightness; mc, moisture content; ΔE*, total color change.

## 4 DISCUSSION

### 4.1 Color, moisture and pH of cricket flour samples during storage

In all samples, L* (lightness) was greatest on day 0 and lowest on day 60 in storage. Similarly, a* was highest on day 0 and lowest on day 30, though having a non‐significant difference among samples and storage duration, and the samples' tendency to yellowness (b*) declined over the course of storage, with no significant variation between samples. This could show the impact of long‐term storage on flour color. Cricket flour had lower lightness values than reported for other kinds of flours (Deepa & Umesh Hebbar, 2017; Gerardi et al., 2022; Uchechukwu‐Agua et al., 2015). Differences in color values L* a* b* of flour from previous studies were attributed to the color of the raw materials and other ingredients that are used in the flour blend. In this study, ginger and garlic extracts were used to preserve crickets rather than powder or paste, and this did not significantly impact the color. According to Kaur et al. (2013), differences in the color characteristics of flours could be attributed to differences in the colored pigments of the flours, level of oxidation which in turn depend on the composition of the flour.

During storage, there was a general decrease in lightness, a tendency to redness, and yellowness of the flour. This could be attributed to the chemical changes that occur during storage as a result of the interaction of the nutrients in the flour with the environment. The total color change (ΔE*) was highest in untreated flour samples on day 60 (11.71 ± 0.55) and lowest in garlic (8.59 ± 1.06) and sodium benzoates (8.86 ± 0.35) preserved flour. This indicates the potential of the garlic and sodium benzoate to reduce chemical reactions taking place in food as a result of the interaction of nutrients in the flour with the environment. Deterioration of color during storage may also indicate a loss of nutritional content as a result of the auto‐oxidation reactions (Uchechukwu‐Agua et al., 2015). Color features in a color space with their respective indices are widely used in food product inspection (Siswantoro, 2019). Specific applications of color measurements in food inspection include grading, detection of anomalies or damage, detection of specific content, and evaluation of color changes. The function of traditional inspection, which is frequently expensive, labor‐intensive, and unreliable, is now largely replaced by automated visual inspection systems for food products using computer vision (Patel et al., 2012). In the food industry, color is a crucial quality element that affects consumers' tastes and choices (Pathare et al., 2013).

During the 60 days of ambient storage in polyethylene bags, all samples showed a non‐significant rise in moisture content. The storage of TARO flour and paste revealed the same pattern (Mbofung, 2010). Additionally, after 90 days of storage in polyethylene bags, flour samples from infant food maintained a constant moisture level (Forsido et al., 2021). The ability of the packaging material to reduce moisture exchange with the storage environment was associated with the moisture stability. Polyethylene bags have a water vapor permeability of 86 g μm/m^2^ day kPa at 27°C and 100% RH, which is lower than reported for other kinds of packaging materials at the same storage conditions (Wang et al., 2018). Since flour is a product that is moisture‐sensitive, long‐term storage in less permeable packaging material is a requirement for increasing shelf life and maintaining product quality (Li et al., 2018). Moisture levels greater than 12% permit microbial growth and product degradation (Kaur et al., 2013). Therefore, the low moisture content reported in this study suggests that ambient storage conditions (23 ± 2°C, 60% RH) are sufficient to maintain the quality attributes and sustain the shelf life of cricket flour past 60 days of storage.

The pH of the cricket flour in this investigation varied significantly between samples and over the storage period, rising gradually from day 0 to day 30 and drastically from day 30 to day 60, this trend was attributed to pattern of formation of the chemical substance responsible for influencing pH in the sample. This is consistent with a pattern that was previously observed for meat‐based products in storage (Kakimov et al., 2019; Mgbemere et al., 2011). In this study, the likely accumulation of slightly alkaline components due to autolytic breakdown or microbial activity was thought to cause the increase in pH from 6.39 to 6.90 ± 0.03 of cricket flour during the 60‐day storage period, as in agreement with previous reports (Liu et al., 2010). The pH values, however, were comparable to those found in freshly ground pearl millet, wheat, and maize flour (Goyal et al., 2017). It is asserted that flours with pH levels in these ranges are acceptable (Apea‐Bah et al., 2011).

### 4.2 Microbial profile and safety of cricket flour samples

The microbial population of samples decreased as storage time increased; with spice extract‐treated and sodium benzoate‐treated samples exhibiting a greater reduction. This was attributed to components in the extract being able to inhibit certain key metabolic processes in the microbes hence reducing their ability to reproduce The results were similar to those that were reported for several brands of cricket powder on the retail market in the United States of America. However, lower counts of 1.98 ± 0.28 and 1.64 ± 0.01 log cfu/g for TVC and yeast, and molds were reported for house cricket (*Acheta domesticus*) flour (Fröhling et al., 2020). The disparity could be attributed to species variation and a difference in the processing conditions in the previous studies. Klunder et al. (2012) reported a significant reduction in TVC from 5.4 log cfu/g on day 0 to a non‐detectable level on days 10–16 of storage of house cricket (*A. domesticus*) flour. At the end of 12 weeks of storage, TVC of ˂6 log cfu/g was reported in cricket (*A. domesticus*) powder, while yeast and molds were not detected (Messina et al., 2019). During the 60 days of storage, this study did not detect *E. coli* or fecal coliforms in the cricket powder. This was attributed to the heat treatment (drying at 105°C for 2 h), which was sufficient to destroy the fecal coliforms during the flour preparation, coupled with hygienic preparation processes. Live crickets are associated with a variety of pathogenic organisms, including fecal coliforms and *E. coli* (Grispoldi et al., 2021). Therefore, their absence from a food product is indicative of quality and safety (Woh et al., 2017). Moreover, the East African standard for insect foods requires ˂1 log cfu/g for ready‐to‐use insect products (KEBS, 2020). The general reduction in the microbial population during the storage period is probably due to the low moisture content resulting in low water activity (Jakab et al., 2020); also the packaging in polyethylene material that limited the interaction of the insect powders with the environment, hence limiting the microbial growth requirements (Forsido et al., 2021).

Cricket flour treated with garlic extracts had the lowest TVC, yeast, and mold counts (1.91 ± 0.00 log cfu/g) at the end of the 60‐day storage period. This was linked to the activity of the garlic antimicrobial compounds such as allicin which is reported to be stronger than ginger in previous studies (Adetunde et al., 2014; El‐Sayed et al., 2017; Panpatil et al., 2013). The relatively high TVC and yeast and molds in the untreated sample is indicative of the effectiveness of the garlic and ginger extracts used in this study to treat the crickets prior to the flour processing. This is in agreement with earlier research on the ability of spice extracts to preserve food (Beristain‐Bauza et al., 2019).

### 4.3 Sensory quality and acceptability cricket flour

Sensory quality is one of the most important features in any food product, including insects. The panelists liked the color of both treated and untreated cricket flour, but the scores dropped as the number of days the flour was stored increased. Food color is affected by chemical, biological, microbial, and physical changes that take place throughout the many stages of food production, handling, processing, and storage (Pathare et al., 2013). In our study, the color of the flour produced by treating crickets with ginger and garlic extracts and processing them into flour did not change noticeably. However, both treated and untreated samples' color ratings were at their lowest on day 60 of storage, suggesting possible biochemical changes such as oxidation during prolonged storage. Color could be the first sensory indication that consumers use to create their expectations of the overall quality of a product (Chonpracha et al., 2020). The mean aroma score of treated and untreated samples was less than 3 (neither liked nor disliked) from 30 to 60 days of storage, receiving the lowest evaluations of all the sensory qualities that were evaluated. The natural aroma of the compounds in the cricket, which were released during the oven drying, was thought to be the source of the aroma of flour. There has been evidence of significant volatile compound variation among insect species (Perez‐Santaescolastica et al., 2022). In a previous study, sensory panelists lowly rated the aroma of oven‐and microwave‐dried locust and silk worm in comparison to the samples that had been freeze‐dried (Mishyna et al., 2020). Thermally treated foods, including insects, exhibit greater concentrations of lipid oxidation compounds and Maillard reaction byproducts (Liceaga, 2021; Lund & Ray, 2017; Perez‐Santaescolastica et al., 2022). Therefore, it is crucial to select the ideal roasting or drying conditions for aroma quality. The drying conditions used in this study could have improved the aroma. In a previous report, the characteristic aroma of roasted insects was shaped by 11 odor‐active compounds, with insect roasted at 160°C exhibiting a characteristic the aroma of baked bacon (Żołnierczyk & Szumny, 2021). The aroma of food allows an initial evaluation of the taste that consumers can expect. However, enzymatic reactions, microbiological growth, or chemical changes in the food product may result in the production of new volatile compounds inside the food package during storage (Han et al., 2018). These changes can be directly related to the sensory quality of the food during storage.

The texture of the treated and untreated cricket flour samples was appreciated by the panelists. Even though the texture rating decreased with storage, its mean score was higher than 3. This qualifies the flour for use in later food applications since texture influences other functional properties of the flour such as solubility, water binding capacity, swelling power, and pasting properties. In earlier research, consumers had a positive response to the textural qualities of foods enriched with cricket flour, including crackers (Akullo et al., 2018; Ardoin et al., 2021; Bartkiene et al., 2022; Duda et al., 2019). The acceptability of the treated and untreated cricket flour samples did not significantly differ. However, after 60 days of storage, a considerable decline was observed. This was attributed to the color and aroma's decreasing acceptability at that time. The same trend was observed when extruded composite flour was stored (Forsido et al., 2021). However, the type of packaging and duration of storage had an impact on how much the score decreased. Acceptability of cricket flour is a pre‐requisite for its subsequent food application. Recent research has reported that cricket flour is acceptable to consumers when blended with other ingredients or in cooperation with other products (Burt et al., 2020; Liceaga, 2021; Simeon et al., 2022; Zebib et al., 2020). In this study, male panelists scored sensory characteristics higher than female panelists, including overall acceptability. This demonstrates that males liked the cricket flour more than females did.

### 4.4 PCA of physiochemical microbial and sensory characteristics

Two components were extracted from the PCA results, which accounted for 89.1% of the variability in the original data. On PC1 and PC2, samples were grouped based on how long they had been in storage. Furthermore, samples of cricket flour treated with spice extracts and samples treated with sodium benzoate grouped together, whereas samples of untreated cricket flour were independent. This is consistent with earlier results, in which PCA of meat stored for varying lengths of time revealed separate clusters based on the length of storage (Arsalane et al., 2017). Additionally, PCA was used to distinguish between fish samples from various storage times based on the volatile chemicals accumulated during storage (Zhao et al., 2021). Both the spice‐treated and sodium benzoate‐treated cricket flour had good color, aroma, and texture right after flour processing, which accounted for the relatively high acceptability at day 0 of storage. While the sensory quality slightly deteriorated after 30 days of storage, this in turn decreased consumer preference. Cricket flour had a high correlation between moisture, pH, and color change after 60 days of storage, indicating that the product's quality had decreased as a result of possible autolytic or microbiological degradation, which explains the product's decreased sensory quality and general acceptability. Compared to the spice‐treated and sodium benzoate‐treated samples, this shift was even more pronounced in the untreated samples. In contrast to the treated samples, the total viable count, yeast, and molds were strongly correlated with the untreated cricket flour. This could explain how the spice extracts influenced microbial multiplication in the cricket flour. Previous studies have shown that spices like garlic and ginger have antimicrobial properties (Abdalla & Abdallah, 2018; Sommano et al., 2016). Considering all the parameters, Day 0 samples were the best, as revealed by the high predicted values.

## 5 CONCLUSION

The color change (Δ*E*), pH, and moisture content of cricket flour increased with the length of storage while the microbial load decreased during storage. When compared, the untreated cricket flour had a higher total microbial load, and yeast and molds compared to the treated, with garlic extract preserved flour having the lowest microbial load. Consumers liked both the spice‐treated and control cricket flour samples, but acceptability significantly declined with storage time. A strong negative correlation existed between the Δ*E*, pH, and moisture content of the cricket flour and the sensory score, while there was a significant positive correlation between sensory scores and overall acceptability. The study showed that using ginger and garlic extracts to treat crickets results in flour that is safe, shelf‐stable, and liked by customers. However, the sensory score and overall acceptance decline with time spent in storage. Therefore, the study concluded that treating crickets with ginger and garlic extracts produces flour that is safe, shelf‐stable and liked by consumers, although sensory score and acceptability are decreased with prolonged storage. Further research is recommended on nutrient, bioactives, anti‐nutrients, composition of spice treated cricket flour to promote its use in the food industry.

## FUNDING INFORMATION

This work was supported by the German Academic Exchange Services (DAAD) through the Regional Universities Forum for Capacity Building in Agriculture (RUFORUM), In‐Country/In‐Region Scholarship Programme #1 under Grant number 1D:57429563; International Foundation for Science (IFS) #2 under Grant number I‐3‐E‐6597‐1.

## CONFLICT OF INTEREST STATEMENT

The authors report there are no competing interests to declare.

## ETHICS STATEMENT

This study does not involve any human or animal testing.

## Supporting information


**Data S1:** Supporting information

## Data Availability

There is no additional data to this manuscript and supplementary material.
