# Supplementary material for: Color, pH, microbiological, and sensory quality of crickets (Gryllus bimaculatus) flour preserved with ginger and garlic extracts
Source: Food Sci Nutr. 2023 Mar 2;11(6):2838–51. doi: 10.1002/fsn3.3262 (PMC10261783; doi:10.1002/fsn3.3262)
Supplement: Supplementary file 1 — Data S1: Supporting information [file FSN3-11-2838-s001.docx]

Summplementary material 1

Table 8. Proportion of principal components

| Component | Eigenvalue | Difference | Proportion | Cumulative |
| --- | --- | --- | --- | --- |
| Comp1 | 9.2763 | 7.8550 | 0.7730 | 0.7730 |
| Comp2 | 1.4213 | 0.6972 | 0.1184 | 0.8915 |
| Comp3 | 0.7241 | 0.4777 | 0.0603 | 0.9518 |
| Comp4 | 0.2464 | 0.1299 | 0.0205 | 0.9724 |
| Comp5 | 0.1165 | 0.0331 | 0.0097 | 0.9821 |
| Comp6 | 0.0834 | 0.0211 | 0.0070 | 0.9890 |
| Comp7 | 0.0623 | 0.0282 | 0.0052 | 0.9942 |
| Comp8 | 0.0341 | 0.0107 | 0.0028 | 0.9971 |
| Comp9 | 0.0234 | 0.0172 | 0.0020 | 0.9990 |
| Comp10 | 0.0062 | 0.0023 | 0.0005 | 0.9995 |
| Comp11 | 0.0040 | 0.0023 | 0.0003 | 0.9999 |
| Comp12 | 0.0017 | . | 0.0001 | 1.0000 |

Supplementary material 2

Table 9. Showing the PCA parameters and the predicted values

| **Samples** | **PCA variables** | | | | | | | | | | | | **Predicted values** | | |
| --- | --- | --- | --- | --- | --- | --- | --- | --- | --- | --- | --- | --- | --- | --- | --- |
|  | **Mc** | **pH** | **L*** | **a*** | **a*** | **ΔE** | **TVC** | **Y&m** | **Color** | **Aroma** | **Texture** | **Accept** | **PC1** | **PC2** | **Id** |
| C+G0 | 1.47 | 6.42 | 31.9 | 4.7 | 16.1 | 0 | 3.62 | 3.03 | 3.67 | 3.59 | 3.89 | 3.77 | 3.616727 | -0.48943 | 3.070928 |
| C+G30 | 2.34 | 6.46 | 26.67 | 3.6 | 10 | 2.29 | 3.19 | 2.3 | 3.41 | 2.74 | 3.55 | 3.36 | -0.30753 | 0.03268 | -0.26231 |
| C+G60 | 2.87 | 6.9 | 25.07 | 3.23 | 8.97 | 10.05 | 2.5 | 2.06 | 2.74 | 2.75 | 3.3 | 3.25 | -3.16103 | -0.71311 | -2.83565 |
| C+Ga0 | 1.21 | 6.4 | 32 | 4.97 | 16.3 | 0 | 3.07 | 2.65 | 3.84 | 3.44 | 4.07 | 3.72 | 3.800794 | -1.36499 | 3.114147 |
| C+Ga30 | 1.9 | 6.41 | 25.43 | 4.3 | 9.53 | 2.09 | 2.54 | 2.63 | 3.31 | 2.81 | 3.47 | 3.24 | -0.1771 | -0.41281 | -0.20843 |
| C+Ga60 | 2.93 | 6.85 | 26.07 | 3.73 | 10.23 | 8.59 | 1.91 | 1.91 | 2.68 | 2.4 | 3.13 | 2.94 | -3.52552 | -1.31598 | -3.23183 |
| C+GGa0 | 1.42 | 6.39 | 31.2 | 4.73 | 16.57 | 0 | 3.18 | 2.93 | 3.72 | 3.54 | 4.11 | 3.77 | 3.732486 | -0.97096 | 3.107294 |
| C+GGa30 | 2.14 | 6.45 | 26.83 | 4 | 10.27 | 2.27 | 3.37 | 2.81 | 3.31 | 2.92 | 3.43 | 3.29 | 0.059678 | 0.413315 | 0.106684 |
| C+GGa60 | 2.88 | 6.84 | 25.8 | 2.63 | 9.67 | 9.04 | 2.45 | 2.06 | 2.62 | 2.69 | 3.15 | 3.02 | -3.61329 | -0.50232 | -3.19978 |
| C+SB0 | 1.44 | 6.43 | 31.07 | 4.4 | 14.4 | 0 | 3.72 | 2.56 | 3.67 | 3.41 | 4.1 | 3.72 | 3.175631 | -0.53161 | 2.682857 |
| C+SB30 | 2.33 | 6.45 | 25.57 | 3.8 | 10 | 2.18 | 3.34 | 2.26 | 3.4 | 2.72 | 3.59 | 3.5 | -0.12288 | 0.078326 | -0.09613 |
| C+SB60 | 2.82 | 6.75 | 24.67 | 2.87 | 8.5 | 8.86 | 2.56 | 1.91 | 2.7 | 2.79 | 3.11 | 3.08 | -3.42499 | -0.48578 | -3.0343 |
| C0 | 1.48 | 6.42 | 32.9 | 4.33 | 15.07 | 0 | 4.8 | 4.66 | 3.72 | 3.52 | 3.9 | 3.59 | 3.97544 | 2.12326 | 3.729244 |
| C30 | 2.53 | 6.43 | 25.97 | 3.47 | 9 | 2.87 | 4.23 | 3.89 | 3.31 | 2.52 | 3.52 | 3.43 | -0.13609 | 2.526708 | 0.217852 |
| C60 | 3.02 | 6.81 | 23.3 | 2.83 | 8.63 | 11.71 | 3.74 | 2.91 | 2.55 | 2.4 | 3.26 | 2.83 | -3.89232 | 1.612691 | -3.16059 |

Mc= moisture content, L* = Lightness, a* = redness/greenness, b*= yellowness/blueness, ΔE* =Total color change
